# Combining H-FABP and GFAP increases the capacity to differentiate between CT-positive and CT-negative patients with mild traumatic brain injury

**DOI:** 10.1371/journal.pone.0200394

**Published:** 2018-07-09

**Authors:** Linnéa Lagerstedt, Juan José Egea-Guerrero, Alejandro Bustamante, Ana Rodríguez-Rodríguez, Amir El Rahal, Manuel Quintana-Diaz, Roser García-Armengol, Carmen Melinda Prica, Elisabeth Andereggen, Lara Rinaldi, Asita Sarrafzadeh, Karl Schaller, Joan Montaner, Jean-Charles Sanchez

**Affiliations:** 1 Department of Specialities of Internal Medicine, Faculty of Medicine, University of Geneva, Geneva, Switzerland; 2 NeuroCritical Care Unit, Virgen del Rocío University Hospital, Seville, Spain; 3 Neurovascular Research Laboratory, Vall d’Hebron Institute of Research (VHIR), Universitat Autònoma de Barcelona, Barcelona, Spain; 4 Division of Neurosurgery, Geneva Neuroscience Center, Department of Clinical Neurosciences, Geneva University Hospitals, Geneva, Switzerland; 5 Intensive Medicine Unit, Hospital Universitario La Paz, idiPAZ, Department of Medicine, Universidad Autónoma de Madrid, Madrid, Spain; 6 Neurosurgical Department, Neuroscience Unit, Hospital Universitari Germans Trias i Pujol, Badalona, Spain; 7 Emergency Department, Hospital de Tortosa Verge de la Cinta, Tortosa, Spain; 8 Emergency Center, Geneva University Hospitals, Geneva, Switzerland; 9 Department of Surgery, Geneva University Hospitals, Geneva, Switzerland; 10 Stroke Research Programme, IBiS/Hospital Universitario Virgen del Rocío/CSIC/University of Seville, Seville, Spain; 11 Department of Neurology, Hospital Universitario Virgen Macarena, Seville, Spain; Case Western Reserve University, UNITED STATES

## Abstract

Mild traumatic brain injury (mTBI) patients may have trauma-induced brain lesions detectable using CT scans. However, most patients will be CT-negative. There is thus a need for an additional tool to detect patients at risk. Single blood biomarkers, such as S100B and GFAP, have been widely studied in mTBI patients, but to date, none seems to perform well enough. In many different diseases, combining several biomarkers into panels has become increasingly interesting for diagnoses and to enhance classification performance. The present study evaluated 13 proteins individually—H-FABP, MMP-1, MMP-3, MMP-9, VCAM, ICAM, SAA, CRP, GSTP, NKDA, PRDX1, DJ-1 and IL-10—for their capacity to differentiate between patients with and without a brain lesion according to CT results. The best performing proteins were then compared and combined with the S100B and GFAP proteins into a CT-scan triage panel. Patients diagnosed with mTBI, with a Glasgow Coma Scale score of 15 and one additional clinical symptom were enrolled at three different European sites. A blood sample was collected at hospital admission, and a CT scan was performed. Patients were divided into two two-centre cohorts and further dichotomised into CT-positive and CT-negative groups for statistical analysis. Single markers and panels were evaluated using Cohort 1. Four proteins—H-FABP, IL-10, S100B and GFAP—showed significantly higher levels in CT-positive patients. The best-performing biomarker was H-FABP, with a specificity of 32% (95% CI 23–40) and sensitivity reaching 100%. The best-performing two-marker panel for Cohort 1, subsequently validated in Cohort 2, was a combination of H-FABP and GFAP, enhancing specificity to 46% (95% CI 36–55). When adding IL-10 to this panel, specificity reached 52% (95% CI 43–61) with 100% sensitivity. These results showed that proteins combined into panels could be used to efficiently classify CT-positive and CT-negative mTBI patients.

## Introduction

Biomarkers have been intensively studied for their potential as diagnostic tools in cases of mild traumatic brain injury (mTBI): to allow accurate diagnosis, improve patient management speeds and reduce medical costs.[1, 2] mTBI is diagnosed from its clinical symptoms and a Glasgow Coma Scale (GCS) score between 13 and 15.[3] Determining whether patients have a trauma-induced brain lesion requires a head CT scan.[4, 5] However, CT scans are widely overused, as only 10% of mTBI patients who undergo one will be diagnosed with a brain lesion.[6, 7] In an attempt to reduce the high numbers of CT scans performed, several proteins have been investigated as potential triage markers. These include S100 calcium binding protein B (S100B) and glial fibrillary acidic protein (GFAP) both astrocyte damage markers, heart fatty acid binding protein (H-FABP) an intracellular vascular and brain fatty-acid transporter and interleukin 10 (IL-10) an anti-inflammatory protein. [8–21] The wide range of biomarker types investigated so far can be explained by the complex pathophysiological nature of TBI.[1] The mechanical forces of a trauma can lead to cell damage due to the shearing, tearing and stretching of neurons, axons, glial and blood vessels, and this damage will further induce biochemical alterations such as excitotoxicity, necrosis and apoptosis, oxidative stress and inflammation.[4, 22] Similar pathophysiological alterations can also be observed in other acute brain injury disorders, such as stroke.[22] A wide range of proteins, of different origins and from different pathways, have been studied as biomarkers for stroke diagnostics and prognostics.[23] However, despite the similarities between these conditions, the performances of several of these biomarkers have never been studied in relation to mTBI.

Regardless of the condition, single markers have been shown to lack the specificity and sensitivity necessary for their use as diagnostic tools in clinical settings.[4, 24] Indeed, to be a useful biomarker, the sensitivity needs to be very high in order to safely discharge patients without performing a CT scan. [7, 10] Furthermore, high specificity would reduce the harmful radiation exposure and it has been shown that a 10% CT scan reduction could save $20 million annually.[7, 18] Despite this, most mTBI biomarker research has been performed with single biomarkers. Combining different markers into a panel has been suggested in order to increase diagnostic performance.[1, 24] Panels have previously been shown to significantly increase diagnostic performance in several different diseases, e.g. sleeping sickness, aneurysmal subarachnoid haemorrhage and lung cancer, and in differentiating between mTBI patients and controls.[25–31] Furthermore, it has been suggested that combinations of different clinical parameters, such as age and even biomarker types, e.g. inflammation proteins and brain damage proteins, can improve classification.[24, 31]

We hypothesized that previously discovered stroke biomarkers could, due to the similarity in pathophysiology, be used as biomarkers also in mTBI patients. A total of 13 proteins—H-FABP, the matrix metalloproteinases 1, 3 and 9 (MMP-1, MMP-3 and MMP-9 respectively), the vascular and intravascular cell adhesion molecules (VCAM and ICAM respectively), IL-10, the inflammatory proteins serum amyloid A (SAA) and C-reactive protein (CRP), the oxidative stress proteins glutathione S-transferase pi (GSTP), nucleoside diphosphate kinase A (NKDA) and peroxiredoxin 1 (PRDX1) and the parkinson disease protein 7 (PARK7/DJ-1)—were chosen.[10, 16, 23, 32–37] These proteins were investigated for their individual performances as CT-scan triage biomarkers in mTBI patients with a GCS score of 15 and at least one clinical symptom. The best-performing proteins were then compared with the two most studied mTBI biomarkers: S100B and GFAP. Furthermore, we hypothesized that proteins combined into panels could increase the capacity to predict CT scan results.

## Materials and methods

### Study population

Patients were recruited from three different European sites: Geneva, Seville and Barcelona. The study was approved by the local ethics committees: Geneva’s Human Research Ethics Committee (CER: 12–194 / NAC 12–074); Barcelona’s Hospital Universitari Vall d’Hebron Ethics Committee (PR_AG_195–2012); and Seville’s Virgen del Rocío University Hospital Institutional Review Board (2012PI/120). Prior to inclusion, written informed consent was obtained from each patient or their legal representatives. Detailed inclusion and exclusion criteria have been specified elsewhere.[10] For inclusion, patients were diagnosed with mTBI and had a GCS score of 15 and at least one additional clinical symptom (vomiting or nausea, loss of consciousness, amnesia, an equilibrium disorder or a headache) and age above 14 years old. Each patient had a blood sample taken at hospital admission ≤ 6 h post trauma and a CT scan was performed within 24h post trauma. Exclusion criteria were no CT scan, no clinical symptoms, GCS score below 15, pregnancy and no signed informed consent form.

### Analysis of proteins

Heparin plasma samples, collected in Geneva, and serum samples, collected in Seville and Barcelona, were centrifuged and stored at -80°C. The NDKA protein was analysed using a custom-made ELISA previously described in detail elsewhere.[35] The remaining proteins were quantified using commercial immunoassay kits (S1 Table) according to their manufacturers’ recommendations.

### Statistical analysis

Due to the study population’s heterogeneity, both in terms of the samples (plasma/serum) and assays used, biomarker results were normalised using their medians as correction factors. The study population was divided into two larger two-centre cohorts. Cohort 1 was used for discovery and verification; Cohort 2 was used for validation. Within each cohort, patients were dichotomised into CT-negative and CT-positive groups for statistical analyses. Non-parametric tests were used hence all proteins were non-parametrically distributed, as indicated by the Kolomogorov-Smirnov test (p < 0.05). The non-parametric Mann–Whitney U test was used to establish differences between the groups, and Fisher’s exact test and the Chi-squared test were used for statistical analyses of the clinical data. Statistics were calculated using IBM SPSS software, version 24.0 (SPSS Inc., Chicago, IL, USA). The performances of individual proteins were tested using receiver operating characteristic (ROC) curves using TIBCO Spotfire S+^®^ version 8.2 software (TIBCO Software Inc., Palo Alto, CA, USA).

### Panel development

Panel experiments were performed using the Panelomix toolbox, which uses the iterative combination of biomarkers and thresholds (ICBT) method.[38] In brief, Panelomix selects cut-offs for each biomarker or clinical parameter to create the optimal panel performance. The panels’ maximum size was set to four parameters. The panel’s performance was investigated when sensitivity reached 100%.

## Results

### Single-marker performances

Cohort 1 included 132 patients, of whom 21 were CT-positive (16%). The two most common findings detected using CT scan were subarachnoid haemorrhage (43%) and skull fracture (38%) (Table 1). Most patients were male, had loss of consciousness or amnesia as clinical symptoms and had an isolated brain trauma (Table 2). Falls and traffic accidents were the most common mechanisms of injury in both CT-positive and CT-negative patients. The only significantly different clinical factor was age, with CT-positive patients being older than CT-negative patients (p < 0.01).

**Table 1:** Brain lesion findings in CT-positive mTBI patients. Patients may have suffered from more than one lesion type, thus making the total percentage exceed 100%.

CT scan results	n (%)
**Epidural haemorrhage**	2 (10)
**Subdural haemorrhage**	5 (24)
**Subarachnoid haemorrhage**	9 (43)
**Intracerebral haemorrhage**	4 (19)
**Contusion with haemorrhage**	6 (29)
**Skull fracture**	8 (38)

**Table 2:** Cohort 1 mTBI patient characteristics ≤ 6 h post trauma.

Cohort 1	CT-negative	CT-positive	p-value^†^
**CT scan**, n (%)	111 (84)	21 (16)	
**Time, trauma to blood**, (min)			0.367^‡^
Mean (SD)	195 (86)	177 (100)	
Median (IQR)	195 (120–255)	160 (83–255)	
**Age**, mean (SD)	46 (21)	63 (24)	**0.003**^‡^
**Male**, n (%)	82 (74)	14 (67)	0.496
**Symptoms**, n (%)			
Amnesia	68 (61)	15 (71)	0.377
Loss of consciousness	93 (84)	20 (95)	0.307
Nausea/vomiting	27 (24)	6 (29)	0.680
Headache	55 (50)	7 (33)	0.172
**Mechanism of injury**, n (%)			
Traffic accident	30 (27)	8 (38)	0.304
Fall	51 (46)	10 (48)	0.888
Assault	15 (14)	2 (10)	1
Sports	3 (3)	-	1
Others	8 (7)	1 (5)	1
NA	4 (4)	-	
**Isolated trauma**, n (%)	89 (81)	15 (71)	0.378
**NA**, n (%)	1 (1)	-	

^†^Chi-square test or Fisher’s exact test

^‡^Mann–Whitney U test

SD: standard deviation, IQR: interquartile range, NA: not available

Thirteen proteins—H-FABP, MMP-1, MMP-3, MMP-9, VCAM, ICAM, IL-10, SAA, CRP, GSTP, NKDA, PRDX1 and DJ-1—were evaluated individually for their CT-scan result prediction capacity. Analyses were performed on the first 62 patients recruited in the 132 mTBI patients of Cohort 1, of whom were 48 CT-negative and 14 CT-positive. Among the 13 biomarkers, only the H-FABP and IL-10 proteins were found at significantly higher levels in CT-positive patients than in CT-negative patients (p < 0.05) (Table 3). Each protein’s individual performance was established at 100% sensitivity, and specificity reached 33% for H-FABP and 27% for IL-10.

**Table 3:** Blood concentrations of 13 biomarkers in 48 CT-negative and 14 CT-positive mTBI patients, and the individual specificity performances.

Protein	CT- median (IQR)	CT+ median (IQR)	P-value	Cut-off	% SE (95% CI)	% SP (95% CI)
**IL-10**	0.1 (0.06–1.2)	0.5 (0.2–1.1)	**0.000**	0.06	100 (100–100)	27.1 (14.6–39.6)
**H-FABP**	2.8 (1.8–4.4)	4.4 (2.4–7.6)	**0.030**	2.0	100 (100–100)	33.3 (20.8–47.9)
**VCAM**	586.6 (438.6–724.0)	667.0 (536.8–861.3)	0.145	359.2	100 (100–100)	12.5 (4.2–22.9)
**GSTP**	211.9 (83.3–469.2)	224.6 (142.1–609.8)	0.354	42.1	100 (100–100)	10.4 (2.1–18.8)
**CRP**	1970.5 (687.5–3977.7)	2875.7 (676.5–15578.9)	0.363	132.4	100 (100–100)	4.2 (0.0–10.4)
**SAA**	1259.3 (911.8–2555.5)	2489.2 (563.2–9058.8)	0.439	279.2	100 (100–100)	8.3 (2.1–16.7)
**DJ-1**	382.3 (96.6–1867.9)	468.8 (163.3–5071.6)	0.501	50.8	100 (100–100)	16.7 (6.3–27.1)
**PRDX1**	86.0 (40.8–211.8)	94.0 (56.0–231.3)	0.643	23.5	100 (100–100)	6.3 (0.0–14.6)
**NDKA**	14.0 (8.0–38.0)	16.0 (9.0–36.8)	0.775	-	100 (100–100)	-
**ICAM**	428.3 (345.7–507.3)	418.0 (332.0–550.9)	0.814	-	100 (100–100)	-
**MMP-3**	17.8 (11.0–28.6)	17.1 (10.7–26.4)	0.866	-	100 (100–100)	-
**MMP-1**	19.5 (11.7–45.7)	20.0 (9.9–33.2)	0.946	5.5	100 (100–100)	10.4 (2.1–18.8)
**MMP-9**	200.7 (122.5–370.7)	198.9 (122.2–358.9)	0.987	505.0	100 (100–100)	8.3 (2.1–16.7)

All protein concentrations are shown in ng/mL except for IL-10, which is in pg/mL.

IQR: interquartile range, SE: sensitivity, SP: specificity

**Fig 1:**
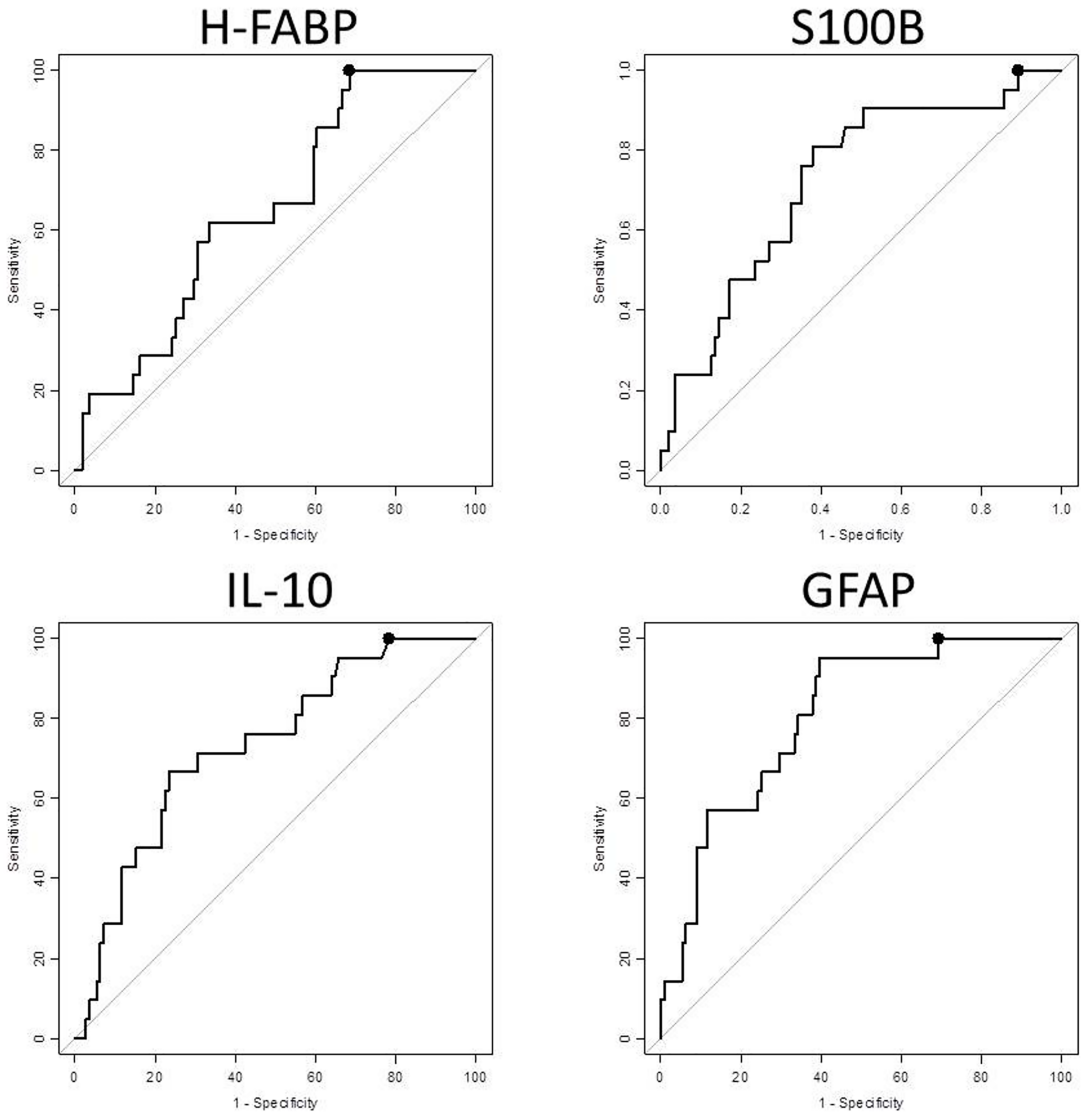
The proteins performances at classifying mTBI CT-positive and CT-negative patients. Performance was investigated at 100% sensitivity, and the specificity (dots) reached 31.5% for H-FABP (95% CI 23.4–39.6; cut-off: 1.99 ng/mL), 10.8% for S100B (95% CI 5.4–17.1; cut-off: 0.06 ug/L), 21.6% for IL-10 (95% CI 14.4–28.8; cut-off: 0.12 pg/mL) and 30.6% for GFAP (95% CI 22.5–39.6; cut-off: 97.31 pg/mL).

The best performing proteins—H-FABP and IL-10—were further compared to both S100B and GFAP for their individual capacities to predict CT scan results across all the mTBI patients in Cohort 1. All four proteins were found at significantly higher levels in CT-positive patients than in CT-negative patients (p < 0.05). The S100B, IL-10 and GFAP proteins reached specificities of 11%, 22% and 31%, respectively, when sensitivity was at 100%. The best performing protein was H-FABP; it reached 32% specificity and 100% sensitivity (Fig 1).

### Combination of markers in panels

**Fig 2:**
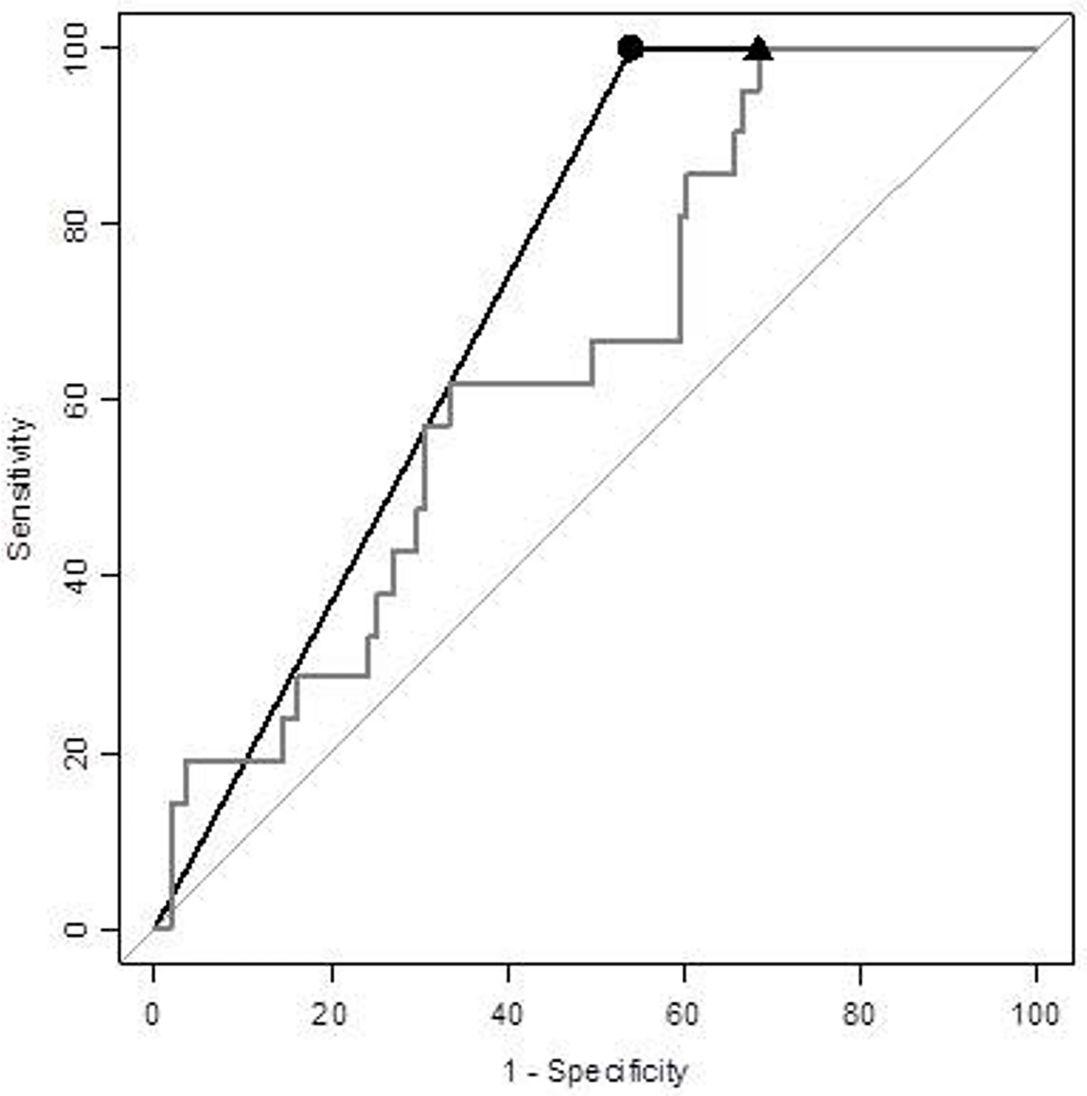
The best two-biomarker panel, combining H-FABP and GFAP, at correctly classifying mTBI CT-positive and CT-negative patients compared to the individual best-performing protein: H-FABP. Performance was investigated at 100% sensitivity, and the panel’s specificity reached 46% (dot, cut-off: 2 parameters, black), compared to the best-performing single marker, H-FABP, which reached 32% specificity (triangle, cut-off: 1.99 ng/mL, grey).

In an attempt to increase specificity, these four proteins were further analysed when combined in panels. All two-protein panels, except for one, showed increased specificity over the best-performing single molecule’s performance: H-FABP’s, at 32%. The best performing panel using two biomarkers was the combination of H-FABP and GFAP, which reached 46% specificity (Table 4 and Fig 2).

**Table 4:** The best-performing panel combinations for Cohort 1.

Panel size	Biomarkers (cut-off)	n CT-	n CT+	Panel cut-off	% SE (95% CI)	% SP (95% CI)
**2 parameters**	**H-FABP** (1.99) **GFAP** (97.31)	111	21	2	100 (100–100)	**45.9** (36.0–55.0)
**3 parameters**	**H-FABP** (1.99) **GFAP** (97.3) **IL-10** (0.12)	111	21	3	100 (100–100)	**52.3** (43.2–61.3)
**4 parameters**	**GFAP** (97.3) **H-FABP** (1.99) **S100B** (0.061) **IL-10** (0.12)	111	21	4	100 (100–100)	**55.9** (46.8–64.9)

The protein concentrations are for H-FABP are shown in ng/mL, S100B in ug/L and IL-10 and GFAP in pg/mL.

Several studies have highlighted age as a risk factor for brain lesions and, as stated above, age was significantly different and therefore included to the combinations as an additional parameter.[39, 40] The panel combination of a single protein and age only increased specificity by a maximum of 3% over H-FABP alone (S2 Table). An increase in specificity was observed when panel size was expanded. Indeed, when combining three proteins, H-FABP + GFAP + IL-10, specificity reached 52%, which was 6% better than the best two-parameter panel. The best-performing panel included all four proteins, reaching a specificity of 56% at a sensitivity of 100% (Table 4 and S2 Table).

### Panel validation

Independent Cohort 2 was used for panel validation on 109 patients, of whom 17 (16%) were CT-positive. The cohort had similar clinical data to Cohort 1, however, age was not significantly different between CT-positive and CT-negative patients (S3 Table). At the single-molecule performance level, when sensitivity was 100%, H-FABP remained the single best-performing protein (S4 Table).

The best-performing panels found using Cohort 1, were validated using Cohort 2. The three panels had a variation in specificity of < 4% between the two cohorts. The panel including all four proteins only increased specificity by 2% in comparison to the best three-parameter panel: H-FABP + GFAP + IL-10. (Table 4 and S5 Table). The most stable combination was H-FABP and GFAP, with only 1% difference in specificity between the cohorts, ranging from 45%–46%, which was 14% higher than the performance observed with a single best molecule: H-FABP (Fig 2).

## Discussion

The present multicentre study evaluated 13 biomarkers, all previously investigated in stroke patients, for their capacity to correctly classify CT-positive and CT-negative mTBI patients with a GCS score of 15 and at least one clinical symptom. Among the 13 biomarkers, the H-FABP and IL-10 proteins were the best-performing single markers, and these were then further compared and combined with the well-studied S100B and GFAP markers. H-FABP was the best-performing single marker, but when combined with GFAP, overall performance increased from 32% to 46% specificity, with sensitivity at 100%.

The four proteins—S100B, GFAP, H-FABP and IL-10—have all previously been identified for their potential to differentiate CT-positive and CT-negative mTBI patients, confirming the results found here.[10, 16, 19, 41] The proteins have been shown to be released from or leak out of different types of injured cells. Indeed, S100B and GFAP leak from injured astrocytes, H-FABP leaks from endothelial cells and neuron cell bodies, whereas Il-10 is expressed by monocytes and macrophages.[8, 11–14, 42, 43] The difficulty, for all biomarker research for brain injuries, is to know if the measured proteins really originate from the brain injury. The four proteins measured here have also been shown to be expressed in cells outside CNS or to be increased after orthopaedic trauma.[2, 44–46] Previously, H-FABP performance has been shown higher in isolated mTBI patients compared to those suffering from multiple traumas.[10] This suggests that at least a part of the H-FABP measured originate from the CNS. Furthermore, elevated levels of H-FABP in blood samples after a stroke have been shown to originate from the CNS.[47] The possiblity to measure the presence of brain derived H-FABP in the blood may be, as suggested for S100B and GFAP, due to blood brain barrier damage or through the glymphatic system.[2]

Independent of origin, single biomarkers have been indicated to not display sufficient performance to be turned into diagnostic tests.[17] Combinations of markers, i.e. panels, have been shown to increase diagnostic performance when combining proteins of diverse origins and different pathways.[24] Diagnostic combinations created using GFAP, H-FABP, S100B and IL-10 resulted in an efficient panel constituted of H-FABP and GFAP and reaching 46% specificity and 100% sensitivity. The panel result was confirmed using a second, independent, two-centre cohort. It is interesting to note that even though the individual performance of the biomarkers varied in each cohort, the panel was found to be stable across both cohorts and it increased specificity by 14% compared to the best single biomarker.

Combinations of biomarkers as a CT-scan triage tool have previously been shown to result in high prediction capacity. A combination including matrix metalloproteinase-2 (MMP-2), CRP and creatine kinase B type (CKBB) held an excellent AUC of 96%. However, in contrary to the results shown here, the combination was presented as a model performed using a likelihood ratio approach.[48] This approach highly complicated to be implemented in a clinical setting. This strategy gives an overall probability of the combination performance without cut-off values for each marker that indicate which patients have to be considered as CT-positive. The panels obtained using PanelomiX have all individual cut-offs for each marker and also a panel cut-off indicating how many of the markers need to be classified as positive in order for the panel to be classified as positive. The use of this tool greatly increases the potential clinical application and feasibility. It would be very interesting to investigate the combination model proposed by Sharma et al. using the PanelomiX tool in order to compare both approaches.

The number of parameters included in a panel may vary from two to dozens. However, the test’s cost-effectiveness must be maintained and over-fitting should be avoided. Our panel composed of H-FABP and GFAP reached a specificity of 45%–46% with a sensitivity of 100%. Adding a third protein was shown here to be even more efficient to avoid CT-scans. However, the costs of measuring all the possible parameters may exceed the costs of using a CT scan to diagnose patients with a significant brain lesion. Other large multicentre studies will be needed to reduce the risk of overfitting and to investigate the panel’s cost-effectiveness.

Another interesting field, requiring exploration, would be the use of the panel in a point-of-care testing (POCT) kit. POCT can greatly reduce time spent analysing and deciding, and it can also be used closer to the patients, e.g. at local medical practices.[49] Several companies are currently developing these kinds of tools. The panel, and potentially its corresponding POCT, could also be interesting tools for outcome measurements. Indeed, both H-FABP and GFAP have been shown to be predictive biomarkers for poor outcome in severe TBI patients.[37, 50] It would, therefore, be interesting to evaluate their individual and combined prediction capacities of poor outcome in mTBI patients.

The present study had certain limitations. Patients originated from three different European sites and only mTBI patients with a GCS score of 15, plus one additional clinical symptom, were included. These strict inclusion criteria reduced the number of CT-negative patients, as patients with a GCS score of 15 but no symptoms were excluded from the study. Furthermore, different samples were collected (plasma or serum), and different kits were used to measure the proteins, depending on the cohort. These differences meant that a clear cut-off level for each protein could not be precisely set up, and this would need additional investigation. However, the study’s results were highly reproducible from cohort to cohort, suggesting an extremely low over-fitting bias.

## Conclusion

This multicentre study showed that combining the measurement of the H-FABP and GFAP proteins into a single panel test could be used to efficiently classify CT-positive and CT-negative patients. The panel significantly outperformed H-FABP alone, the best-performing individual molecule, by reaching 46% specificity and 100% sensitivity. By adding IL-10 to the panel, overall performance reached 52% specificity at 100% sensitivity.

## Supporting information

S1 Table Summary of the immunoassays used in this study. (DOCX)

S2 Table All panel combinations involving the different individually significant H-FABP, GFAP, S100B and IL-10 proteins and age, in Cohort 1. (DOCX)

S3 Table Cohort 2 mTBI patients’ characteristics < 6 h post-trauma. (DOCX)

S4 Table Performance of single biomarkers in Cohort 2 with sensitivity reaching 100%. (DOCX)

S5 Table The best performing panels in Cohort 1 were validated using Cohort 2 with sensitivity reaching 100%. (DOCX)

S1 Dataset The 13 markers raw data and patient information. (XLSX)

S2 Dataset Cohort 1 raw data and patient information. (XLSX)

S3 Dataset Cohort 2 raw data and patient information. (XLSX)
