# Supplementary material for: Combining H-FABP and GFAP increases the capacity to differentiate between CT-positive and CT-negative patients with mild traumatic brain injury
Source: PLoS One. 2018 Jul 9;13(7):e0200394. doi: 10.1371/journal.pone.0200394 (PMC6037378; doi:10.1371/journal.pone.0200394)
Supplement: S1 Table — (DOCX) [file pone.0200394.s001.docx]

**S1 Table. Summary of the immunoassays used in this study.**

| **Protein** | **Patient recruitment site** | **Kit reference** | **L****OQ** | **Units** | **Manufacturer** |
| --- | --- | --- | --- | --- | --- |
| CRP | Geneva | K15198D | 27.6–49600 | pg/mL | Meso Scale Diagnostics, Rockville, MD, USA |
| DJ-1 | Geneva | K151THD | 24–100000 | pg/mL | Meso Scale Diagnostics, Rockville, MD, USA |
| GFAP | Geneva/Seville/Barcelona | F211M | 63.0–500000 | pg/mL | Meso Scale Diagnostics, Rockville, MD, USA |
| GSTP | Geneva | prototype | 7–30000 | pg/mL | Meso Scale Diagnostics, Rockville, MD, USA |
| H-FABP | Geneva | HK402 | 102–25000 | pg/mL | Hycult Biotech, Uden, The Netherlands |
| H-FABP | Seville/Barcelona | K151HTD | 137–100000 | pg/mL | Meso Scale Diagnostics, Rockville, MD, USA |
| ICAM | Geneva | K15198D | 6.4–32700 | pg/mL | Meso Scale Diagnostics, Rockville, MD, USA |
| IL-10 | Geneva/Seville/Barcelona | K151QUD | 0.298–233 | pg/mL | Meso Scale Diagnostics, Rockville, MD, USA |
| MMP-1 | Geneva | K15034C | 24–100000 | pg/mL | Meso Scale Diagnostics, Rockville, MD, USA |
| MMP-3 | Geneva | K15034C | 24–100000 | pg/mL | Meso Scale Diagnostics, Rockville, MD, USA |
| MMP-9 | Geneva | K15034C | 122–500000 | pg/mL | Meso Scale Diagnostics, Rockville, MD, USA |
| NDKA | Geneva | Custom made | 0.78–100 | ng/mL | Custom made |
| PRDX-1 | Geneva | KA0536 | 0.5–32 | ng/mL | Abnova, Taiwan |
| S100B | Geneva/Barcelona | EZHS100B-33K | 2.74–2000 | pg/mL | Millipore, Billerica, MA, USA |
| S100B | Seville | Elecsys 2010 immunoassay system | 0.005–39 | µg/L | Roche Diagnostics, Germany |
| SAA | Geneva | K15198D | 54.0–138000 | pg/mL | Meso Scale Diagnostics, Rockville, MD, USA |
| VCAM | Geneva | K15198D | 37.6–32000 | pg/mL | Meso Scale Diagnostics, Rockville, MD, USA |

LOQ: limit of quantification
