# Supplementary material for: Combining H-FABP and GFAP increases the capacity to differentiate between CT-positive and CT-negative patients with mild traumatic brain injury
Source: PLoS One. 2018 Jul 9;13(7):e0200394. doi: 10.1371/journal.pone.0200394 (PMC6037378; doi:10.1371/journal.pone.0200394)
Supplement: S2 Table — (DOCX) [file pone.0200394.s002.docx]

**S2 Table. All panel combinations involving the different individually significant H-FABP, GFAP, S100B and IL-10 proteins and age, in Cohort 1.**

| **Panel size** | **Markers**  (cut-off) | **n CT-** | **n CT+** | **Panel cut-off** | **% SE** (95% CI) | **% SP** (95% CI) |
| --- | --- | --- | --- | --- | --- | --- |
| **2-parameter panels** | H-FABP (1.99)  GFAP (97.3) | 111 | 21 | 2 | 100 (100–100) | **45.9** (36.0–55.0) |
|  | GFAP (97.3)  IL-10 (0.12) | 111 | 21 | 2 | 100 (100–100) | 45.0 (36.0–55.0) |
|  | H-FABP (1.99)  IL-10 (0.12) | 111 | 21 | 2 | 100 (100–100) | 42.3 (33.3–51.4) |
|  | GFAP (97.3)  S100B (0.061) | 111 | 21 | 2 | 100 (100–100) | 36.9 (27.9–45.9) |
|  | H-FABP (1.99)  S100B (0.061) | 111 | 21 | 2 | 100 (100–100) | 36.0 (27.0–45.9) |
|  | IL-10 (0.12)  S100B (0.061) | 111 | 21 | 2 | 100 (100–100) | 28.8 (20.7–37.8) |
|  | H-FABP (1.99)  age (17.5) | 111 | 21 | 2 | 100 (100–100) | 35.1 (26.1–44.1) |
|  | GFAP (97.3)  age (17.5) | 111 | 21 | 2 | 100 (100–100) | 35.1 (27.0–44.1) |
|  | S100B (0.15)  age (42.0) | 111 | 21 | 1 | 100 (100–100) | 30.6 (22.5–38.7) |
|  | IL-10 (0.12)  age (17.5) | 111 | 21 | 2 | 100 (100–100) | 25.2 (17.1–33.3) |
| **3-parameter panels** | H-FABP (1.99)  GFAP (97.3)  IL-10 (0.12) | 111 | 21 | 3 | 100 (100–100) | **52.3** (43.2–61.3) |
|  | H-FABP (1.99)  GFAP (97.3)  S100B (0.06) | 111 | 21 | 3 | 100 (100–100) | 49.5 (39.6–58.6) |
|  | H-FABP (1.99)  IL-10 (0.12)  S100B (0.06) | 111 | 21 | 3 | 100 (100–100) | 45.9 (36.9–55.0) |
|  | GFAP (97.3)  IL-10 (0.12)  S100B (0.061) | 111 | 21 | 3 | 100 (100–100) | 45.9 (40.5–59.5) |
|  | H-FABP (1.99)  GFAP (97.3)  age (17.5) | 111 | 21 | 3 | 100 (100–100) | 49.5 (40.5–58.6) |
|  | GFAP (97.3)  IL-10 (0.12)  age (17.5) | 111 | 21 | 3 | 100 (100–100) | 48.6 (39.6–58.6) |
|  | H-FABP (1.99)  IL-10 (0.12)  age (17.5) | 111 | 21 | 3 | 100 (100–100) | 45.0 (35.1–54.1) |
|  | IL-10 (0.12)  S100B (0.15)  age (42.0) | 111 | 21 | 2 | 100 (100–100) | 45.0 (36.0–54.1) |
|  | H-FABP (1.99)  S100B (0.15)  age (42) | 111 | 21 | 2 | 100 (100–100) | 44.1 (35.1–54.1) |
|  | GFAP (97.3)  S100B (0.15)  age (42.0) | 111 | 21 | 2 | 100 (100–100) | 44.1 (35.1–53.2) |
| **4-parameter panels** | GFAP (97.3)  H-FABP (1.99)  S100B (0.061)  IL-10 (0.12) | 111 | 21 | 4 | 100 (100–100) | **55.9** (46.8–64.9) |
|  | GFAP (97.3)  H-FABP (1.99)  IL-10 (0.12)  age (17.5) | 111 | 21 | 4 | 100 (100–100) | 55.0 (45.9–64.0) |
|  | H-FABP (1.99)  S100B (0.15)  IL-10 (0.12)  age (42.0) | 111 | 21 | 3 | 100 (100–100) | 53.2 (44.1–62.2) |
|  | GFAP (97.3)  H-FABP (1.99)  S100B (0.061)  age (17.5) | 111 | 21 | 4 | 100 (100–100) | 53.2 (43.2–62.2) |
|  | GFAP (97.3)  S100B (0.061)  IL-10 (0.12)  age (17.5) | 111 | 21 | 4 | 100 (100–100) | 52.3 (43.2–61.3) |

The cut-off concentrations for H-FABP are in ng/mL, for GFAP and IL-10 in pg/mL, and for S100B in ug/L.

SE: sensitivity, SP: specificity
