# Supplementary material for: Combining H-FABP and GFAP increases the capacity to differentiate between CT-positive and CT-negative patients with mild traumatic brain injury
Source: PLoS One. 2018 Jul 9;13(7):e0200394. doi: 10.1371/journal.pone.0200394 (PMC6037378; doi:10.1371/journal.pone.0200394)
Supplement: S3 Table — (DOCX) [file pone.0200394.s003.docx]

**S3 Table. Cohort 2 mTBI patients’ characteristics < 6 h post-trauma.**

| **Cohort 2** | **CT-negative** | **CT-positive** | **p-value^†^** |
| --- | --- | --- | --- |
| **CT scan**, n (%) | 92 (84) | 17 (16) |  |
| **Time trauma to blood**, (min) |  |  | 0.553^‡^ |
| Mean (SD) | 175 (83) | 187 (105) |  |
| Median (IQR) | 163 (106–244) | 210 (65–274) |  |
| **Age**, mean (SD) | 51 (22) | 60 (22) | 0.122^‡^ |
| **Male**, n (%) | 55 (60) | 12 (71) | 0.400 |
| **Symptoms**, n (%) |  |  |  |
| Amnesia | 61 (66) | 14 (82) | 0.189 |
| Loss of consciousness | 74 (80) | 13 (77) | 0.745 |
| Nausea/vomiting | 13 (14) | 3 (18) | 0.713 |
| Headache | 21 (23) | 1 (6) | 0.186 |
| **Mechanism of injury**, n (%) |  |  |  |
| Traffic accident | 23 (25) | 5 (29) | 0.765 |
| Fall | 36 (39) | 2 (12) | **0.030** |
| Assault | 15 (16) | 3 (18) | 1 |
| Sports | 5 (5) | 1 (6) | 1 |
| Others | 11 (12) | 5 (29) | 0.127 |
| NA | 2 (2) | 1 (6) |  |
| **Isolated trauma**, n (%) | 67 (73) | 14 (82) | 0.552 |

^†^Chi-square test or Fisher’s exact test

^‡^Mann–Whitney U test.

SD: standard deviation, IQR: interquartile range, NA: not available
