# Supplementary material for: Combining H-FABP and GFAP increases the capacity to differentiate between CT-positive and CT-negative patients with mild traumatic brain injury
Source: PLoS One. 2018 Jul 9;13(7):e0200394. doi: 10.1371/journal.pone.0200394 (PMC6037378; doi:10.1371/journal.pone.0200394)
Supplement: S4 Table — (DOCX) [file pone.0200394.s004.docx]

**S4 Table. Performance of single biomarkers in Cohort 2 with sensitivity reaching 100%.**

| **Marker** | **n CT-** | **n CT+** | **Cut-off** | **% SE** (95% CI) | **% SP** (95% CI) |
| --- | --- | --- | --- | --- | --- |
| H-FABP | 92 | 17 | 1.96 | 100 (100–100) | **35.9** (26.1–45.7) |
| GFAP | 92 | 17 | 117.26 | 100 (100–100) | **21.7** (13.0–30.4) |
| IL-10 | 92 | 17 | 0.12 | 100 (100–100) | **12.0** (5.4–18.5) |
| S100B | 92 | 17 | 0.04 | 100 (100–100) | **9.8** (4.3–16.3) |

The cut-off concentration for H-FABP is in ng/mL, for GFAP and IL-10 in pg/mL, and for S100B in ug/L.

SE: sensitivity, SP: specificity
