# Supplementary material for: Combining H-FABP and GFAP increases the capacity to differentiate between CT-positive and CT-negative patients with mild traumatic brain injury
Source: PLoS One. 2018 Jul 9;13(7):e0200394. doi: 10.1371/journal.pone.0200394 (PMC6037378; doi:10.1371/journal.pone.0200394)
Supplement: S5 Table — (DOCX) [file pone.0200394.s005.docx]

**S5 Table. The best performing panels in Cohort 1 were validated using Cohort 2 with sensitivity reaching 100%.**

| **Panel size** | **Biomarkers**  (cut-off) | **n CT-** | **n CT+** | **Panel cut-off** | **% SE** (95% CI) | **% SP** (95% CI) |
| --- | --- | --- | --- | --- | --- | --- |
| **2 parameters** | H-FABP (1.96)  GFAP (117.26) | 92 | 17 | 2 | 100 (100–100) | 44.6 (34.8–54.4) |
| **3 parameters** | H-FABP (1.96)  GFAP (117.26)  IL-10 (0.12) | 92 | 17 | 3 | 100 (100–100) | 50.0 (39.1–60.9) |
| **4 parameters** | GFAP (117.26)  H-FABP (1.96)  S100B (0.04)  IL-10 (0.12) | 92 | 17 | 4 | 100 (100–100) | 52.2 (42.4–62.0) |

The cut-off concentrations for H-FABP are in ng/mL, for GFAP and IL-10 in pg/mL, and for S100B in ug/L.

SE: sensitivity, SP: specificity
